# The accuracy of gypsum casts obtained from the disinfected extended-pour alginate impressions through prolonged storage times

**DOI:** 10.1186/s12903-021-01649-2

**Published:** 2021-06-09

**Authors:** Rania A. Sharif, Khalid M. Abdelaziz, Najla M. Alshahrani, Fatimah S. Almutairi, Mohrah A. Alaseri, Hoda L. Abouzeid, Mohamed F. A. Elagib

**Affiliations:** 1 Department of Prosthodontics, College of Dentistry, King Khalid University, Abha, Saudi Arabia; 2 Department of Restorative Dental Sciences, College of Dentistry, King Khalid University, Abha, Saudi Arabia; 3 King Khalid University, Khamis Mushait, Abha, 62458 Saudi Arabia; 4 Department of Periodontics and Community Dental Sciences, College of Dentistry, King Khalid University, Abha, Saudi Arabia

**Keywords:** Alginate, Disinfection, Detail reproduction, Dimensional stability, Gypsum cast, Storage, Extended-pour

## Abstract

**Background:**

Manufacturers of the extended-pour alginates claimed their dimensional stability through prolonged storage. No data confirmed the ability of these materials to maintain their dimensions and the reproduced oral details following their chemical disinfection. Therefore, this study evaluated the dimensional stability and surface detail reproduction of gypsum casts obtained from disinfected extended-pour alginate impressions through different storage time intervals.

**Methods:**

Two hundred and forty three hydrocolloid impressions were made from one conventional (Tropicalgin) and two extended-pour (Hydrogum 5 and Chromaprint premium) alginates. These impressions were subjected to none, spray and immersion disinfection before their storage in 100% humidity for 0, 72 and 120 h. The dimensional stability and the surface detail reproduction were indirectly evaluated under low angle illumination on the resulted gypsum casts. At α = 0.05, the parametric dimensional stability data were analyzed using One-Way ANOVA and Tukey’s comparisons, while the nonparametric detail reproduction data were analyzed using KrusKal Wallis and Mann–Whitney's tests.

**Results:**

All gypsum casts exhibited a degree of expansion; however, the recorded expansion values did not differ between test categories (*P* > 0.05). Generally, casts obtained from spray-disinfected impressions showed lower detail accuracy (*P* < 0.05). Immersion-disinfected extended-pour alginates produced casts with better detail accuracy following 120 h of storage (*P* < 0.05).

**Conclusion:**

All alginates materials offer comparable cast dimensions under different testing circumstances. Extended-pour alginates offer casts with superior surface details following their immersion disinfection and 120 h of storage. Spray-disinfection using 5.25% sodium hypochlorite affects the surface details of casts obtained from conventional and extended-pour alginates adversely.

## Background

Obtaining successful dental prostheses requires accurate reproduction of soft and hard oral tissues. Accordingly, selection of both impression and cast materials seems critical for optimum biological, functional, and esthetical treatment outcomes [1, 2]. Regardless the modern digital approaches, regular dental impressions are still the most reliable method to obtain gypsum casts with an acceptable degree of precision [3, 4]. The cost effective and easily manipulated irreversible hydrocolloids are one of the most frequently used impression materials in everyday dental practice [5, 6]. However, the dimensional instability in response to the syneresis and imbibition phenomena is considered the main drawback of the conventional version of the alginates [7]. These inherited phenomena are obviously dependent on impression’s ambient storage condition and time. Knowing the former fact mandates the immediate or even early pouring of the irreversible hydrocolloid impressions in gypsum [8, 9].

In some instances, the process of immediate/early pouring could relatively be impossible especially if the impression is planned to be transferred to a dental laboratory. Accordingly, the extended-pour alginates have been developed with the ability to maintain the dimensions of the impressions stable through the extended storage time intervals [8, 10–12]. Alginate alternative with polyvinyl siloxane additives (siliconized alginates) had also been marketed with the privilege of maintaining the impression dimensions through the prolonged storage (100 + h) in addition to the possibility of re-pouring the gypsum casts [13]. Although, some researchers [14] strongly supported the former characteristics, others [15] indicated acceptable dimensional inaccuracy in comparison to the value announced in the American National Standards Institute/American Dental Association (ANSI/ADA) Specification 19.

On the other hand, there are strong recommendations to rinse and disinfect all kinds of impressions after their removal from the patient mouth. This procedure helps get rid of the adhered saliva, blood and microorganisms and accordingly minimize the chances of cross-contamination [16, 17]. Depending on the nature of the impression materials, decontamination of impressions could be achieved using different sterilization and disinfection procedures [18–21]. However, the hydrophilic nature of alginates usually allows higher adsorption of microorganisms onto the impression's body and surfaces [22]. Some researchers [23, 24] had accordingly developed the self-disinfecting alginates and others used solutions of some chemical disinfectants for mixing alginate powder. On the other hand, immersion disinfection of alginate impression could offer a solution for the formerly presented dilemma although it is usually associated with significant values of dimensional changes in comparison to the spray disinfection process [25–29]. Truly, through the last decade, many studies evaluated the dimensional changes in the non-disinfected as well as the disinfected extended-pour alginates [30–32], but only few of them had the concern to assess the combined effect of impression disinfection and the subsequent prolonged storage on the reproduced dimensions and surface details.

Therefore, this study aimed to evaluate the dimensional stability and surface detail reproduction of gypsum casts obtained from immersion and spray-disinfected extended-pour alginate impressions through different storage time intervals. The null hypothesis accordingly was that none of the utilized impression disinfection and storage protocols will have adverse effects on dimensions and surface details of the resulting gypsum casts.

## Methods

**Fig. 1:**
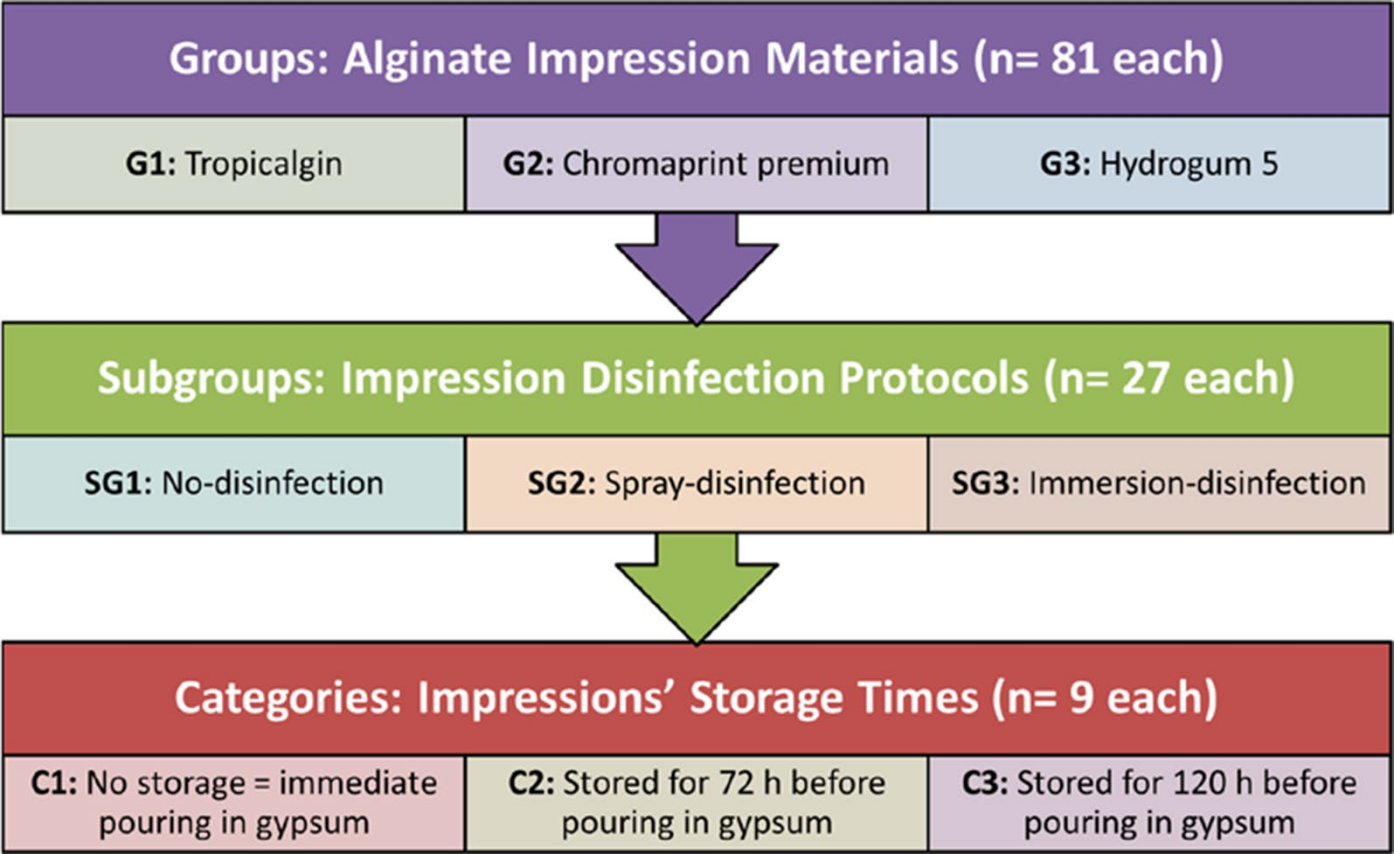
Classification of test specimens

A total of 243 irreversible hydrocolloid impressions of a metal test block were made in custom made trays using one conventional (Tropicalgin, Zhermak, SpA, Badia Polesine, Italy) and two extended–pour (Chromaprint premium; Coltene Whaledent AG, Alstatten, Switzerland and Hydrogum 5; Zhermak, SpA, Badia Polesine, Italy) fast-set alginates. The post‑hoc power (OSP = 0.934) was calculated to assure the reliability of the selected sample size. Names, description and manufacturers of materials used in this study are listed in Table 1, while the classification of test specimens is briefly described in Fig. 1.

**Table 1:** Materials used

Material	Description	Manufacturer
Tropicalgin	Conventional irreversible hydrocolloid impression material *Composition:* potassium alginate, calcium sulfate and a few other elements derived from brown algae	Zhermak, SpA, Badia Polesine, Italy
Chroma print Permium	Extended-pour irreversible hydrocolloid impression material *Composition:* Potassium alginate, diatomite, calcium sulfate, magnesium oxide, tetra sodium pyrophosphate, potassium fluotitanate, PEG, pigment. Ethanol, phenolphthalein and flavor	Coltene Whaledent AG, Alstatten, Switzerland
Hydrogum 5	Extended-pour irreversible hydrocolloid impression material *Composition:* Potassium alginate, diatomaceous earth, calcium sulfate, trisodium phosphate, triaminofunctional silane	Zhermak, SpA, Badia Polesine, Italy
Durone IV	Type IV gypsum cast material (Dental Stone)	Dentsply, Rio de Janeiro, Brazil
Clorox	5.25% Sodium hypochlorite solution	Abodawood, Jeddah, Saudi Arabia
Glutaron	2% Glutaraldehyde solution	Rio Química Ltda, Rio de Janeiro, Brazil

### Fabrication of the test block

**Fig. 2:**
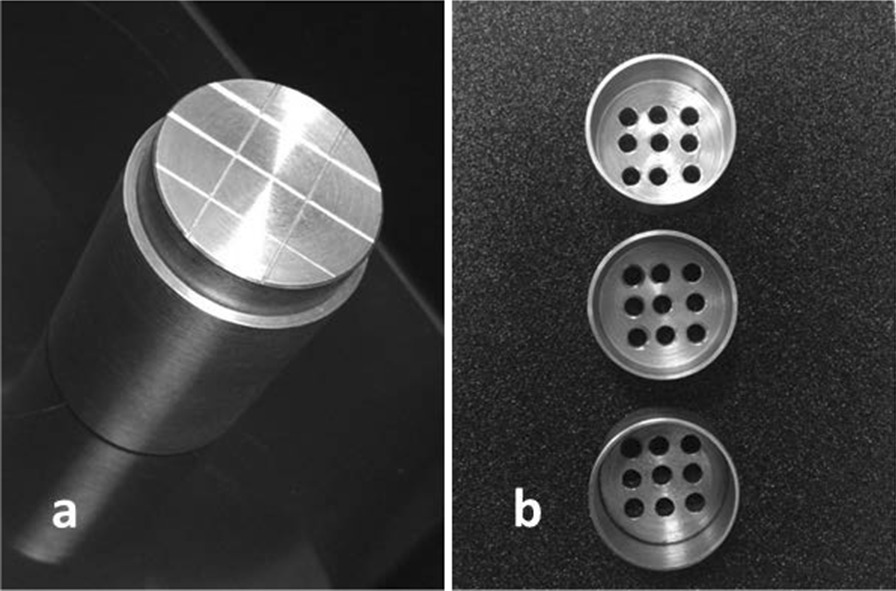
The custom-made **a** metal test block and **b** metal special tray

The metal test block, 3 cm in diameter, was constructed by the aid of CNC lathe machine according to ADA specification No. 25. The top surface of this block was prepared with three parallel straight grooves (0.025, 0.050 and 0.075 mm in width and 60° peak angles) spaced 7.5 mm apart. Another two horizontal 0.050 wide grooves spaced 15 mm apart were also prepared intersecting the first ones at 90° (Fig. 2a). Several perforated custom-made metal trays were also prepared with 1 mm intaglio clearance around the constructed test block (Fig. 2b).

### Making alginate impressions

**Fig. 3:**
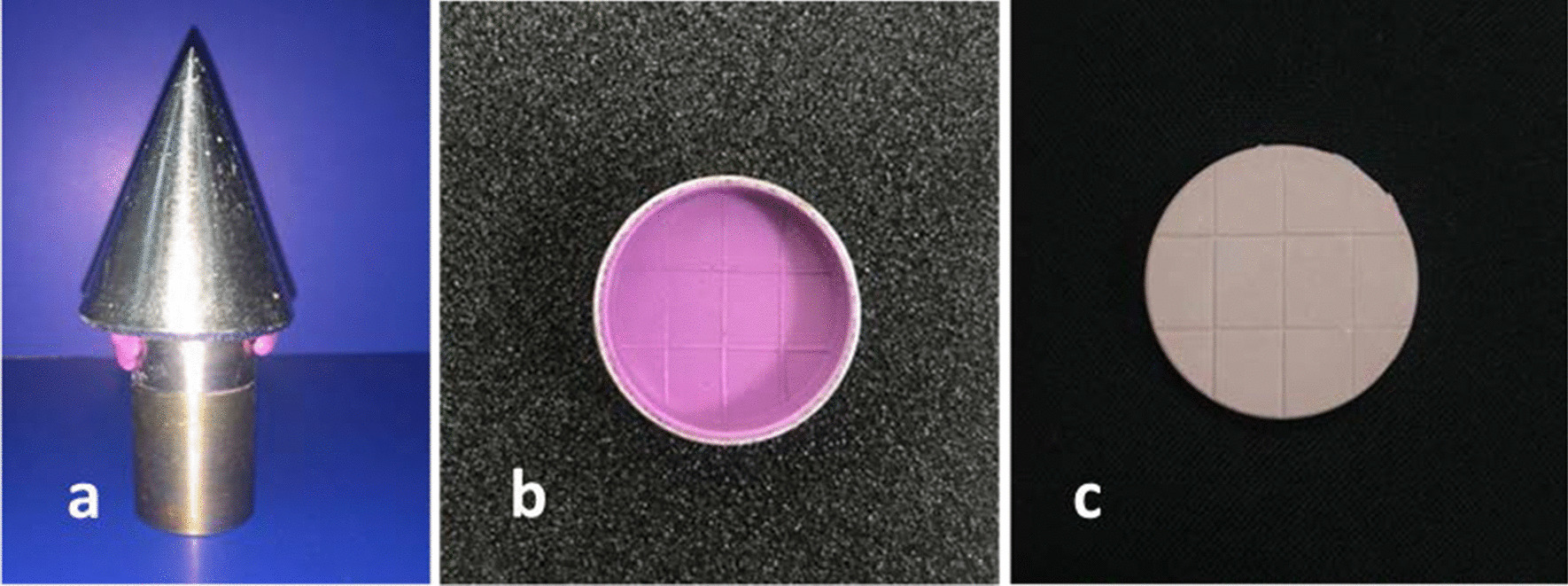
Procedures of impression and cast making; **a** impression making, **b** released impression and **c** the resulted gypsum cast

Impressions of the test block were made in three groups (n = 81 each) according to the type of impression material used. In group 1 (G1), the manufacturer’s recommended L/P ratio of the conventional alginate (Tropicalgin) was hand-mixed in a rubber bowl. The mixture was stirred in one direction using a plaster spatula for 45 s against the bowl walls to achieve air bubble-free, homogeneous mix with a uniform color. The mixed material was loaded into the custom-made metal trays and hand pressed against the test block until metal-to-metal contact is achieved. A standard weight of 1 kg was used to help standardize impression thickness and keep the tray in position for 3 min until the complete setting of the material is achieved (Fig. 3a). Groups 2 (G2) and 3 (G3) impressions were made following the previously mentioned procedure using Chromaprint premium (Coltene) and Hydrogum 5 (Zhermak) extended-pour alginates.

### Impression disinfection and storage

The completely set impressions of each group were taken off the test block (Fig. 3b), washed up with tap water and gently air-dried before their disinfection in three different subgroups (n = 27 each). In subgroup 1 (SG1), impressions were left with no disinfection. In subgroup 2 (SG2), impressions were spray disinfected with 5.25% sodium hypochlorite solution (Clorox, Abodawood, Jeddah, Saudi Arabia). Impression surfaces were totally sprayed with the solution then stored with a disinfectant-wet cotton roll in zip-lock plastic bags for 10 min. At the same time, subgroups 3 (SG3) impressions were subjected to immersion disinfection in 2% glutaraldehyde solution (Glutaron, Rio Química Ltda, Rio de Janeiro, Brazil) for 10 min.

The disinfected and non-disinfected impressions were then washed up under running tap water for 1 min and then subjected to prolonged storage in three categories (n = 9 each) before pouring the gypsum casts. Impressions of the first category (C1) were immediately poured in gypsum with no prolonged storage, while other impressions in categories 2 (C2) and 3 (C3) were stored in relative humidity inside zip-lock plastic bags together with wet cotton rolls for 72 and 120 h respectively [33].

### Pouring the gypsum casts

After disinfection, impressions in all categories were washed up under running water for 1 min and air-dried before pouring them in Extra-hard Type IV gypsum material (Durone IV, Dentsply, Petrópolis, Rio de Janeiro, Brazil). The gypsum product powder was hand mixed with a plaster spatula in rubber bowl using 25 ml of tap water for each 100gm of gypsum powder. High speed vibration (Sabilex dental laboratory vibrator, Sabilex de Flexafil SA, Buenos Aires, Argentina) of the mixed material for 30 s helped get rid of most of the entrapped air bubbles. The alginate impression was filled with gypsum mix while subjecting it to high speed vibration to minimize the chances of air bubbles formation within the gypsum cast. The poured impressions were left in air at room temperature for 1 h to ensure complete setting of gypsum casts (Fig. 3c) before taking them out of the impressions.

### Assessing the accuracy of the resulting casts

Both the dimensional stability and the detail reproduction ability of the tested impression materials in each category were indirectly assessed on the resulted gypsum casts according to ANSI/ADA Specifications No. 18 and 19. All measurements were carried out by three evaluators with inter-personal confidence of 77.8% and the mean of their measurements was recorded.

The dimensional accuracy was assessed on the gypsum cast by measuring the distance between the two horizontal grooves at their intersection with the 0.050 mm wide longitudinal groove using a digital caliper (model 500-196-30, Motutoyo, Kanagawa, Japan). The recorded measurements were then compared to the original distance between the same lines on the metal test block. The resulted data were statistically analyzed using both One-Way ANOVA and Tukey’s comparisons at α = 0.05 to stand on the significance of any differences detected between the 27 test categories.

Assessment of the detail reproduction was also performed on the gypsum casts at X10 original magnification using a magnifying lens. Under low angle illumination, the 0.025 mm wide groove was inspected on each cast. The accuracy of the reproduction through the entire length of the groove was then scored as follows: 4; when well-defined, sharp continuous groove was observed, 3; when continuous groove with some loss of sharpness was observed, 2; when loss of groove’s continuity was observed and 1; when there was failure of reproduction of the entire groove [34, 35]. The collected scores were then statistically analyzed using both Kruskal–Wallis test and Mann–Whitney pairwise comparisons at α = 0.05 to detect the significance of any differences detected between test categories.

## Results

The mean dimensional changes and standard deviations of alginate impressions in different test categories (Shown in Fig. 1) are listed in Table 2. The One-Way ANOVA proved the existence of some differences between test categories (*P* = 0.06E−05).

**Table 2:** Mean dimensional changes (mm) in different test categories (n = 9 each)

Disinfection protocols (n = 27 each)	Storage time intervals (n = 9 each)	Impression materials (n = 81 each)		
		G1	G2	G3
SG1	C1	0.12 ± 0.11^A1^	0.19 ± 0.04^A1^	0.11 ± 0.06^A1^
	C2	0.05 ± 0.24^A1^	0.15 ± 0.13^A1^	0.11 ± 0.06^AB1^
	C3	0.11 ± 0.13^A1^	0.14 ± 0.04^A1^	0.25 ± 0.13^AB1^
SG2	C1	0.06 ± 0.09^A1^	0.17 ± 0.02^A1^	0.16 ± 0.12^A1^
	C2	0.20 ± 0.10^A1^	0.17 ± 0.17^A1^	0.33 ± 0.13^B1^
	C3	0.07 ± 0.08^A1^	0.24 ± 0.12^A1^	0.20 ± 0.06^A1^
SG3	C1	0.11 ± 0.10^A1^	0.18 ± 0.11^A1^	0.15 ± 0.14^AB1^
	C2	0.11 ± 0.08^A1^	0.08 ± 0.19^A1^	0.08 ± 0.04^AB1^
	C3	0.18 ± 0.22^A1^	0.15 ± 0.08^A1^	0.22 ± 0.09^AB1^

G1 = Tropicalgin; G2 = Chromaprint premium; G3 = Hydrogum 5; SG1 = No disinfection; SG2 = Spray disinfection; SG3 = Immersion disinfection; C1 = No storage; C2) 72 h of storage and C3) 120 h of storage

Positive (+ve) values of different test categories indicate expansion in alginate impressions

1-Way ANOVA, *P* = 0.06−E05

Within each column, same superscript letters indicate no difference between test categories within the same test group (Tukey’s, *P* > 0.05)

Within each row, same superscript numbers indicate no difference between test groups within the same test category (Tukey’s, *P* > 0.05)

Generally all impression materials exhibited a degree of expansion in all test categories (+ve values, Table 2). However, the recorded expansion values did not differ between test categories of both G1 [Tropicalgin] and G2 [Chromaprint premium] (Tukey’s *P* > 0.05). In G3 [Hydrogum 5], the spray-disinfected impressions poured after 72 h of storage [SG2, C2] showed higher expansion than the immediately-poured, non-disinfected impressions [SG1, C1] (Tukey’s, *P* = 0.01566) as well as those subjected either to none [SG1, C2] or immersion disinfection [SG3, C2] and poured in gypsum after 72 h of storage (Tukey’s, *P* = 0.0169 and 0.002366).

All impressions in different test groups showed no difference in their expansion values under the same disinfection and storage circumstances [Within the same test category]. (Tukey’s, *P* > 0.05).

### Detail reproduction

The mean detail reproduction scores and the standard deviations of alginate impressions in different test categories (Shown in Fig. 1) are listed in Table 3. It is obvious from the data presented in Table 3 that none of the tested materials failed to completely register the details of 0.025 mm-wide groove of the test block (i.e. No records of score 1), but all of them showed the ability to reproduce the details of that groove with different levels of accuracy (Scores 4, 3 and 2). Statistical analysis of those records using the Kruskal–Wallis test indicated some differences between test categories (*P* = 6.25E−16).

**Table 3:** Mean detail reproduction scores in different test categories

Disinfection protocols (n = 27 each)	Storage time intervals (n = 9 each)	Impression materials (n = 81 each)		
		G1	G2	G3
SG1	C1	2.78 ± 0.44^A1^	3.00 ± 0.00^A1^	3.44 ± 0.53^A1^
	C2	2.89 ± 0.60^A1^	2.67 ± 0.50^A1^	3.56 ± 0.53^A2^
	C3	3.00 ± 0.00^A1^	2.33 ± 0.50^B1^	3.33 ± 0.50^A1^
SG2	C1	2.67 ± 0.50^A1^	3.00 ± 0.00^A1^	3.00 ± 0.00^B1^
	C2	2.11 ± 0.33^B1^	2.22 ± 0.44^AB1^	2.11 ± 0.33^C1^
	C3	3.22 ± 0.67^AC1^	2.00 ± 0.00^BC1^	2.33 ± 0.50^C1^
SG3	C1	3.00 ± 0.00^AC1^	2.67 ± 0.50^AB1^	2.78 ± 0.44^B1^
	C2	3.11 ± 0.33^AC1^	2.89 ± 0.33^A1^	3.22 ± 0.83^AB1^
	C3	2.44 ± 0.53^AB1^	3.00 ± 0.00^A2^	3.67 ± 0.50^A3^

G1 = Tropicalgin; G2 = Chromaprint premium; G3 = Hydrogum 5; SG1 = No disinfection; SG2 = Spray disinfection; SG3 = Immersion disinfection; C1 = No storage; (C2) 72 h of storage and (C3) 120 h of storage

Higher scores in different test categories indicate higher accuracy of the reproduced details

Kruskal–Wallis test, *P* = 6.25E−16

Within each column, same superscript letters indicate no difference between test categories within the same test group (Mann–Whitney, *P* > 0.05)

Within each row, same superscript numbers indicate no difference between test groups within the same test category (Mann–Whitney, *P* > 0.05)

In G1 [Tropicalgin], the spray-disinfected impressions [SG2] recorded lower detail reproduction scores at 72 h of storage in comparison to the immediately-poured non-disinfected impressions of the same group (Mann–Whitney, *P* = 0.006635). The non-disinfected impressions [SG1] of G2 [Chromaprint premium] also showed lower detail reproduction scores when poured at 120 h [C3] of storage than the immediately-poured [C1] ones of the same subgroup (Mann–Whitney, *P* = 0.00). In comparison to the non-disinfected, immediately-poured [SG1, C1] impressions of G3 [Hydrogum 5], the spray-disinfected ones [SG2] showed higher detail reproduction scores when immediately-poured in gypsum (Mann–Whitney, *P* = 0.03214) and lower scores when poured either at 72 or 120 h of storage (Mann–Whitney, *P* = 0.0003758 and 0.002054).

All impressions in different test groups showed no difference in their ability to reproduce the details under the same disinfection and storage circumstances [Within the same test category] (Mann–Whitney, *P* > 0.05). However, at 72 h of storage (C2), the G3, SG1 impressions [non-disinfected Hydrogum 5] showed higher detail reproduction scores than the other impressions of G1 [Tropicalgin] and G2 did [Chromaprint premium] (Mann–Whitney, *P* = 0.03296 and 0.005855). Following immersion disinfection and 120 h of storage, G3 impressions also showed the highest detail reproduction scores than did G1 (Mann–Whitney, *P* = 0.00142) and G2 (Mann–Whitney, *P* = 0.004217) which in turn recorded higher scores than did G1 impressions (Mann Whitney, *P* = 0.01241).

## Discussion

Making alginate impressions is a very common procedure in both prosthodontics and orthodontic work. Accuracy of these impressions is required to reproduce dental cast almost have the same dimensions and details of the oral structures [1, 2]. In spite of the ease of material’s manipulation, alginate impressions usually encounter some dimensional changes in response to their polysaccharide nature [5]. This structure makes these materials regularly subject to fluid gain and loss in inherited phenomena known as imbibition and syneresis [7]. Both phenomena always have an adverse effect on impression dimensional stability during either wet or humid storage, therefore pouring alginate impressions immediately after their removal from the patient’s mouth and thorough washing up was strongly recommended [8, 9]. Accordingly, some manufacturers attempted to solve this problem and introduced the extend-pour alginates that could hypothetically offer stable impressions through the prolonged storage times [11, 12].

On the other hand, alginate impressions, due to their polysaccharide nature and the ability to imbibe microorganism in, were documented to have a great role in cross-contamination between dental patients and both clinical and laboratory staffs; however the commonly used impression disinfection methods were found to affect the stability of alginate impressions [22]. Some researchers [16, 17] preferred to spray-disinfect alginate impressions rather than going through immersion-disinfection protocol. They explain their selection by the minimal change of both impression dimensions and details that is usually reported following the spray-disinfection procedures. Others [25–29] reported massive alteration in both impressions’ dimensions and details following immersion disinfection.

Few studies [30–32] assessed the dimensional changes in the extended pour alginate impressions following their disinfection, but most of them ignored to store these impressions after performing the disinfection procedures. In spite of these records, nearly none of the conducted studies evaluated the alteration in dimensions and details transferred from the extended–pour alginate following different disinfection protocols and through the prolonged storage time intervals. Therefore the current study aimed to assess both the dimensional changes and detail reproduction in gypsum casts produced from both spray and immersion-disinfected extended-pour alginate impressions through prolonged storage time intervals reaching up to 120 h. The null hypothesis of this study accordingly was that none of the tested alginate impression disinfection protocols and prolonged storage times would seriously affect the dimensions and the details of the resulting gypsum casts.

To test the drawn null hypothesis, two extended-pour commercially available alginate impression materials were selected in addition to one conventional alginate to be used as a reference. The manufactures of all the materials, including the conventional one, clearly announced on the materials’ outer packages the ability of these materials to maintain their stability on prolonged storage. The tested chemical disinfectants were also selected due to their wide acceptance in alginate impressions disinfection. Both 5.25% NaOCl (sodium hypochlorite) and 2% glutaraldehyde were reported to offer effective spray and immersion disinfection, with minimal adverse effects on alginate impressions when used according to the manufacturer’s instructions [36–39].

In general, results of this study revealed a degree of minimal expansion in gypsum casts obtained in different test categories; but no difference could clearly be related to any of the testing variables (Impression materials, disinfection protocols and pouring storage) (Table 2). The formerly mentioned manufacturers’ announcement and the noticed nonspecific differences in the composition of the tested alginate materials (Table 1) helped confirm the dimensional stability of the tested impression materials regardless the disinfection and storage circumstances they were subjected to. On the other hand, the minimal thickness of alginate impressions (1 mm thick) in addition to the thick and rigid nature of the used stainless steel trays did not allow for a noticeable change in impressions’ dimensions even with the possible existence of the imbibition phenomenon. [40] The higher cast expansion (0.33 ± 0.13 mm) noticed with the spray-disinfected hydrogum 5 impressions when poured at 72 h [G3, SG2, C2] could not be of practical value because the calculated standard deviation seems to be the highest among those of other test category. This situation indicates big differences between readings recorded for each impression of that category.

Results of Amin et al. [41] may support the former suggestion as they reported comparable stability of conventional alginate impressions subjected to 5–10 min of chemical disinfection with the standard ANSI/ADA specification No.18. Porrelli et al. [42] also revealed significant stability of five extended-pour alginates materials including Hydrogum 5 through storage periods extended to 5 days. In agreement, Imbery et al. [43] reported no difference in the accuracy of gypsum casts obtained from either non-stored or 120 h-stored extended-pour alginates. Moreover, results of Sayed and Gangadharappa [44] indicated that storing of the extended-pour alginate impressions in 100% controlled humidity, using the same protocol utilized in this study, offers the ideal environment to maintain their optimal dimensional accuracy. In coincident with the aforementioned studies, the noticed expansion values in all categories came in agreement with those indicated in the ANSI/ADA Specification 19 and could, by this way, be referred to the documented gypsum setting expansion [15, 39]. Powers [45] stated that Type IV gypsum material can exhibit setting expansion range from 0.0 to 0.15 mm in response to the known outward thrusting of the growing gypsum crystals during the setting process; however this value could vary from a product to another. Long time ago, Durr and Novak [26] also reported minimal clinically insignificant amount of linear dimensional changes in gypsum casts obtained from disinfected alginate impressions in comparison to the original master cast.

At the same time, the recorded detail reproduction data of the gypsum casts obtained in different test categories of the current study showed no difference between the tested alginate impression materials. However, only one exception was noticed between the tested materials after immersion-disinfected and 120 h of storage. In this case, Hydrogum 5 (G3) seemed the best to reproduce details followed by Chromaprint premium (G2) and Tropicalgin (G3) respectively (Table 3). The former findings reflected the higher ability of the extended-pour alginates to maintain the recorded details in comparison to the conventional type even after immersion-disinfection and prolonged storage up to 120 h.

Some studies [34, 46] indicated that the compatibility between alginate impression surfaces and the gypsum cast material is sometimes not exist and that it is usually governed by the type of these materials. Moreover, treating alginate surfaces with different chemicals could dramatically affect the quality of the reproduced surface details. The current results, accordingly, indicated comparable compatibility of the tested alginate materials to the utilized gypsum cast material and this could be referred to the almost similar basic composition of the used alginates. The previous explanation could be supported by the X-ray diffraction results of Abdelraouf [47] that indicated only higher calcium/sodium ratio and lower organic and water contents of the extended-pour alginates in comparison to the conventional ones.

Another study [48] stated that the gel like nature of the set alginate allows the adsorption from the fluids in contact with it especially during the disinfection procedure, but the sensible effect of this phenomenon on the accuracy of impressions and the produced cast is greatly influenced by the contact time. Amin et al. [41] confirmed the safety of the usual alginate impressions disinfection approaches. Immersion disinfection for up to 10 min did not significantly influence the surface quality of the resulted gypsum casts. Based on this information, the disinfection procedure probably had no or little responsibility for the differences detected between the tested impression materials poured following immersion disinfection and 120 h of storage, but the noticed difference could be referred to the influence of storage time and conditions. The extended-pour alginates are designed to withstand prolonged storage with the highest possible degree of accuracy, however the conventional alginate could, for shorter time, show the same ability. This obvious limitation could result from the change in the storage environment in response to the possible fade out of the induced storage humidity and the ability of the material to show the influence of this change on the imbibition/syneresis phenomena [45].

On the other hand, casts obtained from the NaOCl spray-disinfected impressions in different test groups (Impression materials) showed alternative detail reproduction scores which are almost lower than those of casts obtained from non-disinfected and immersion-disinfected impression regardless the tested storage times. These findings reflect a degree of adverse effect of disinfection using NaOCl on the quality of the reproduced details. Although, Hutchings et al. [49] reported no difference in the detail reproduction produced from NaOCl spray-disinfected alginate impressions and those rinsed in water. Shambhu et al. [50] agreed that the concentration and age of the sodium hypochlorite solution in addition to the disinfection contact time have great influence on the effectiveness of sodium hypochlorite. In this study, a high concentration (5.25%) of NaOCl was sprayed and remained on the impression surfaces for 10 min. Moreover, NaOCl is known to show a bit of resistance against the short-term washing up with water. The unwashed residue on the impression surfaces could, in turn, show an erosive effect on the surfaces of gypsum in contact [51]. This explanation could support the possible adverse effect of NaOCl on gypsum cast surfaces. Results of Vandewalle et al. [52] came in agreement and indicated that disinfection of alginate impression with 5.25% sodium hypochlorite usually causes some surface deterioration of casts made up of some types of dental stone. Tan et al. [53] also documented an adverse effect of NaOCl disinfection of alginate impression on the details reproduced on the resulted gypsum casts. Amin et al. [41] also reported loss of sharpness of the grooves reproduced on gypsum casts obtained from alginate impressions disinfected with 5.25% NaOCl.

Based on the recorded results of this study, the drawn null hypothesis could be accepted in part, as some significant effects of disinfection and storage were noticed on the detail reproduction of the resulting casts, and the protocols followed in this study could offer the dental practitioners a safe protocol to store alginate impressions for longer period even after their exposure to meticulous disinfection. However, direct testing on full-arch alginate impressions is advisable in further studies. This approach could offer more accurate results considering the influence of gypsum expansion and surface roughness that surely differ from one product to another based on the compositional additives.

## Conclusions

Within the limitations of this in vitro study, the following conclusions could be deduced;

1. All alginate materials offer comparable cast dimensions under different testing circumstances.
2. Extended-pour alginates offer casts with superior surface details following their immersion disinfection and 120 h of storage.
3. Spray-disinfection using 5.25% NaOCl adversely-affects the surface details of casts obtained from conventional and extended-pour alginates.

## Data Availability

Data is available with the authors and the sources of materials used were addressed in Table 1.

## Abbreviations

NaOCl: sodium hypochlorite
ANSI/ADA: American National Standards Institute/American Dental Association
G1: group 1
L/P ratio: liquid powder
G2: group 2
G3: group 3
SG1: subgroup 1
SG2: subgroup 2
SG3: subgroups 3
C1: First category
C2: second category
C3: third category
